# Antiprotozoal Activity of (*E)*-Cinnamic *N*-Acylhydrazone Derivatives

**DOI:** 10.3390/molecules191220374

**Published:** 2014-12-05

**Authors:** Samir Aquino Carvalho, Marcel Kaiser, Reto Brun, Edson Ferreira da Silva, Carlos Alberto Manssour Fraga

**Affiliations:** 1 Instituto de Tecnologia em Fármacos e Farmanguinhos, Fundação Oswaldo Cruz, Rio de Janeiro, RJ 21041-250, Brazil; E-Mails: scarvalho@far.fiocruz.br (S.A.C.); edsonf@far.fiocruz.br (E.F.S.); 2 Swiss Tropical and Public Health Institute (Swiss TPH), Socinstrasse 57, Basel CH-4002, Switzerland; E-Mails: marcel.kaiser@unibas.ch (M.K.); reto.brun@unibas.ch (R.B.); 3 Laboratório de Avaliação e Síntese de Substâncias Bioativas (LASSBio), Programa de Pesquisa em Desenvolvimento de Fármacos, Instituto de Ciências Biomédicas, Universidade Federal do Rio de Janeiro, P.O. Box 68023, Rio de Janeiro, RJ 21941-902, Brazil; 4 Programa de Pós-Graduação em Química, Instituto de Química, Universidade Federal do Rio de Janeiro, Rio de Janeiro, RJ 21949-900, Brazil

**Keywords:** antiprotozoal activity, *Leishmania*, *Trypanosoma*, *N*-acylhydrazone, (*E*)-cinnamic acid derivatives, molecular hybridization

## Abstract

A series of 14 (*E*)-cinnamic *N*-acylhydrazone derivatives, designed through molecular hybridization between the (*E*)-1-(benzo[*d*][1,3]dioxol-5-yl)-3-(4-bromophenyl)prop-2-en-1-one and (*E*)-3-hydroxy-*N*'-((2-hydroxynaphthalen-1-yl)methylene)-7-methoxy-2-naphthohydrazide, were tested for *in vitro* antiparasitic activity upon axenic amastigote forms of *Leishmania donovani* and bloodstream forms of *Trypamosoma brucei rhodesiense*. The derivative (2*E*)-3-(4-hydroxy-3-methoxy-5-nitrophenyl)-*N*'-[(1*E*)-phenylmethylene]acrylohydrazide showed moderate antileishmanial activity (IC_50_ = 6.27 µM) when compared to miltefosine, the reference drug (IC_50_ = 0.348 µM). However, the elected compound showed an excellent selectivity index; in one case it was not cytotoxic against mammalian L-6 cells. The most active antitrypanosomal compound, the derivative (*E*)-*N'*-(3,4-dihydroxybenzylidene)cinnamohydrazide (IC_50_ = 1.93 µM), was cytotoxic against mammalian L-6 cells.

## 1. Introduction

African sleeping sickness and leishmaniasis continue to cause significant public health problems. These parasitic diseases are responsible for a high rate of mortality and morbidity each year in tropical and subtropical countries [1].

African sleeping sickness is caused by the protozoan parasite *Trypanosoma brucei* exclusively in sub-Saharan Africa. The number of reported cases is decreasing and currently at less than 8000 per year but the estimated number is likely to be around 25,000 [2].

Leishmaniasis is a complex not contagious infectious disease that presents diverse clinical and epidemiological characteristics in each geographic area. The main forms of leishmaniasis are—visceral, cutaneous, and mucocutaneous. It is an endemic disease in many parts of the world and its estimated 1.3 million new cases and 20,000 to 30,000 deaths occur annually [3,4]. Drugs currently in use, such as pentavalent antimony compounds, pentamidine, miltefosine or amphotericin B, are inadequate due to their toxicity, lack of efficacy, availability and affordability, and the inability to eliminate all parasite life cycles stages from the host [5].

In recent years, *trans*-cinnamic acid derivatives have attracted much attention due to their antimycobacterial [6], antimalarial [7], leishmanicidal [8] and antimicrobial activity [9]. It is often employed in the design of bioactive substances [10] due to the presence of an α,β-unsaturated carbonyl moiety, which can be considered as a Michael acceptor unit, as the pharmacophoric group.

Recently, we reported the synthesis and trypanocidal profile of new (*E*)-cinnamic *N*-acylhydrazones (NAH). These derivatives were evaluated against both amastigote and trypomastigote forms of *Trypanosoma cruzi* and lead us to identify two compounds that were approximately two times more active than the reference drug, *i.e.*, benznidazole, and present with good selectivity index [11].

*Trypanosoma* sp. and *Leishmania* sp. are protozoan parasites of the order kinetoplastida. Although they’re different kinetoplastid pathogens have a similar genomic organization and cellular structures [12]. This evolutionary resemblance and the previous results obtained by our research group with *T. cruzi* encouraged us to evaluate a series of fourteen of these derivatives upon axenic amastigote forms of *Leishmania donovani* and also against bloodstream forms of *Trypanosoma brucei*.

**Figure 1:**
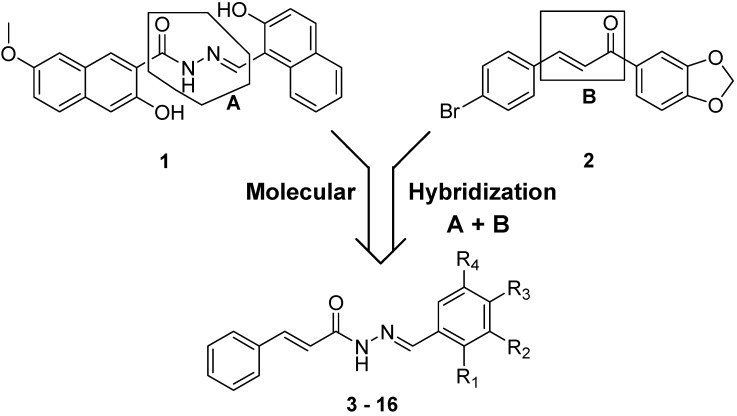
Design concept of cinnamic *N*-acylhydrazone derivatives (**3**–**16**).

The new (*E*)-cinnamic *N*-acylhydrazones were designed by molecular hybridization of two potent inhibitors cysteinyl proteases; the functionalized chalcone **1** [13], a potent inhibitor of cruzipain (IC_50_ = 20 μM), and the bis-naphthyl-*N*-acylhydrazone compound **2** [14], a potent inhibitor of *L. major* cathepsin L-like cysteine protease (IC_50_ = 0.5 μM) (Figure 1), to thereby enhance the inhibitory profile of the target enzyme by offering another electrophilic group capable to interact with the nucleophilic cysteine residue at the active site of the protozoan protease.

## 2. Results and Discussion

The title compounds (**3**–**16**) were prepared in good yields following the synthetic methodology previously described by our research group to access this kind of α,β-unsaturated-*N*-acylhydrazones (see Table 1) [11].

The relative configuration (*E*) for both olefine and imine double bonds was confirmed by X-ray diffraction data obtained for the unsubstituted derivative (**12**) [15] and also for the 4-chlorophenyl (**11**) and 2-hydroxyphenyl (**15**) derivatives [16,17]. After the careful structural characterization by ^1^H- and ^13^C-NMR and mass spectrometry, followed by determination of a purity degree >99% by reversed phase HPLC and elemental analysis [11], all the fourteen cinnamic *N*-acylhydrazone derivatives (**3**–**16**) were submitted to the evaluation of their antiprotozoal profile *in vitro*. 

The antileishmanial, antitrypanosomal and cytotoxic properties of NAH derivatives (**3**–**16**) are shown in Table 1, and their respective IC_50_ were determined. 

**Table 1:** *In vitro* trypanocidal, leishmanicidal and cytotoxic activities of the cinnamic *N*-acylhydrazone derivatives (**3**–**16**). 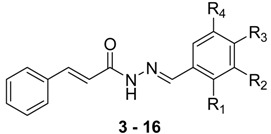

Cpd.	R_1_	R_2_	R_3_	R_4_	*L. donovani* ^a^	*T.b. rhod*. ^b^	Cytot. L-6 Cells ^c^	SI ^d^	SI ^e^
					IC_50_^f^ (μM)				
3	H	OMe	OH	NO_2_	6.27	45.6	>264	>42	>5.8
4	H	OMe	OH	H	21.7	40.8	95.4	4.4	2.3
5	H	OH	OH	H	11.7	1.93	14.0	1.2	7.3
6	H	O-CH_2_-O		H	12.5	89.5	4.3	0.3	0.05
7	H	OMe	OMe	OMe	3.50	49.3	120	34.3	2.4
8	H	H	F	H	24.3	284	>335	>13.8	>1.2
9	H	H	NO_2_	H	10.6	>300	255	24.1	-
10	H	H	OMe	H	13.8	111	>321	23.3	2.9
11	H	H	Cl	H	10.8	121	65.5	6.0	0.5
12	H	H	H	H	43.1	>300	157	3.6	-
13	H	H	OH	H	36.7	82.5	40.4	1.1	0.5
14	H	OMe	OMe	H	31.2	67.6	>290	>9.3	>4.3
15	OH	H	H	H	3.61	6.72	14.9	4.1	2.2
16	H	OH	OMe	H	33.3	80.1	48.1	1.4	0.6
MTS	-	-	-	-	0.348	-	ND	-	-
MLSP	-	-	-	-	-	0.003	ND	-	-
PPT	-	-	-	-	-	-	0.006	-	-

^a^ *Leishmania donovani* (MHOM/ET/67/L82) axenic amastigotes [18]; ^b^ *Trypanosoma brucei rhodesiense* (STIB900) [19,20]; ^c^ Cytotoxicity to L-6 rat myoblast cells [20]; ^d^ Selectivity Index for leishmanicidal activity; ^e^ Selectivity Index for trypanocyde activity; ^f^ IC_50_ values are means of two determinations. ND = Value not determined. Reference drugs: MTS (Miltefosine); MLSP (Melarsoprol); PPT (Podophyllotoxin).

The analysis of antileishmanial results showed that among the three most active compounds (**3**, **7** and **15**) two were trisubstitued. Compound **15** with an *o*-hydroxyphenyl group attached to the imine unit, was one of the most active compounds (IC_50_ = 3.61 μM), although it was cytotoxic to mammal’s cells. This considerable antiprotozoal profile in comparison to the other derivatives could be explained by the ability of the *o*-hydroxybenzylidene *N*-acylhydrazone framework to form an electrophilic quinone methide intermediate [21] that could interact with nucleophilic sites in target enzymes of Leishmania, e.g., cysteine proteases [22,23]. The 3,4,5-trimethoxyphenyl derivative (**3)** and the 5-nitrovanillyl derivative (**7**), both presenting substituents that could act as hydrogen-bond acceptors, showed important inhibitory activity with IC_50_ = 6.27 μM and 3.50 μM, respectively. When compounds (**3**) and (**7**) are compared we could observe the importance of the nitro group for antileishmanial activity, probably to their particular redox properties [24]. When we compared the trisubstituted derivatives (**3**) and (**7**) with the corresponding disubstituted ones (**4**) and (**14**) it is evident the importance of the third substituent, *i.e.*, nitro or methoxy groups respectively, for the leishmanicidal activity. Moreover, mono- or disubstituted *N*-acylhydrazone derivatives (**5**), (**6**), (**9**), (**10**) and (**11**), presenting substituents in the phenyl group attached to the imine unit with different stereoelectronic properties, showed a range of leishmanicidal activity varying from 10.6 to 13.8 μM, supporting the evidence previously discussed about the relevance of a trisubstituted pattern for the best molecular recognition by target bioreceptor. Cinnamic *N*-acylhydrazone derivative (**3**) presented a good selectivity index; in one case it was not toxic against mammalian L-6 cells at the maximum concentration investigated, *i.e.*, 264 μM. 

On the other hand, the most active trypanocyde compound was 3,4-dihydroxyphenyl derivative (**5**), which present an IC_50_ = 1.93 μM. This remarkable activity profile in comparison to the other derivatives could be explained by the pharmacophoric character of the cathecol subunit for the trypanocidal activity [25], which can act as a radical scavenger group interfering in the redox metabolism of the parasite. This result differs from our previously work, where this derivative showed poured activity against *T. cruzi*. The *o*-hydroxyphenyl derivative (**15**) also presented acceptable trypanocidal activity profile, IC_50_ = 6.72 μM, in comparison to the other *N*-acylhydrazone derivatives, and it could be explained by the inhibition of cysteinyl protease, corroborating the *in vitro* trypanocidal activity for this compound described earlier against amastigote and tripomastigote form of *T. cruzi* [11]. Despite to show a good inhibitory potency against *Trypanosoma brucei* strains compounds (**5**) and (**15**) were cytotoxic against L-6 rat myoblast cells, with an IC_50_ of 14.0 and 14.9 μM respectively, not demonstrating an appropriate safety profile for a drug candidate. 

## 3. Experimental Section

### 3.1. Leishmanicidal Activity

Amastigotes of *Leishmania donovani* strain MHOM/ET/67/L82 were grown in axenic culture at 37 °C in SM medium at pH 5.4 supplemented with 10% heat-inactivated fetal bovine serum under an atmosphere of 5% CO_2_ in air. One hundred microliters of culture medium with 10^5^ amastigotes from axenic culture with or without the compound to test were seeded in 96-well microtiter plates. Serial drug dilution assay using seven 3-fold dilutions covering a range from 30 μg/mL to 0.041 μg/mL and determining the 50% inhibition concentration (IC_50_). Each drug was tested in duplicate and each assay was repeated at least once. After 72 h of incubation the plates were inspected under an inverted microscope to assure growth of the controls and sterile conditions. Ten microliters of Alamar Blue (12.5 mg resazurin dissolved in 100 mL phosphate buffered saline) [18] were then added to each well and the plates incubated for another 2 h. Then the plates were read in a Spectramax Gemini XS microplate fluorometer (Molecular Devices Cooperation, Sunnyvale, CA, USA) using an excitation wave length of 536 nm and an emission wave length of 588 nm. Data were analyzed using the software Softmax Pro (Molecular Devices Cooperation, Sunnyvale, CA, USA). Decrease of fluorescence (=inhibition) was expressed as percentage of the fluorescence of control cultures. For the serial drug dilution assay inhibition values were plotted against the drug concentrations and IC_50_ values were calculated from the sigmoidal inhibition curves.

### 3.2. Trypanocidal Activity

Minimum essential medium (50 μL) supplemented according to a known procedure [19] with 2-mercaptoethanol and 15% heat-inactivated horse serum was added to each well of a 96-well microtiter plate. Serial drug dilutions were prepared covering a range from 90 to 0.123 μg/mL. Then 10^4^ bloodstream forms of *T. b. rhodesiense* STIB 900 in 50 μL were added to each well and the plate incubated at 37 °C under a 5% CO_2_ atmosphere for 72 h. Ten microliters of Alamar Blue (containing 12.5 mg resazurin dissolved in 1000 mL distilled water) were then added to each well and incubation continued for a further 2–4 h. The Alamar Blue dye is an indicator of cellular growth and/or viability. The blue, non-fluorescent, oxidized form becomes pink and fluorescent upon reduction by living cells. The plate was then read in a Spectramax Gemini XS microplate fluorometer (Molecular Devices Cooperation, Sunnyvale, CA, USA) using an excitation wavelength of 536 nm and emission wavelength of 588 nm [20]. Fluorescence development was measured and expressed as percentage of the control. Data were transferred into the graphic programme Softmax Pro (Molecular Devices,) which calculated IC_50_ values. Melarsoprol was used as standard. 

### 3.3. Cytotoxicity

Exactly 100 μL RPMI 1640 medium supplemented with 1% L-glutamine (200 mM) and 10% fetal bovine serum containing 4 × 10^4^ L-6 cells (rat skeletal myoblasts) [20] were added to each well of a 96-well microtiter plate. After 24 h, the medium was removed from all wells and replaced by 100 μL of fresh medium containing a 3-fold serial drug dilution covering a range from 200 μg/mL to 0.274 μg/mL, except for the control wells. After 72 h of incubation 10 μL of Alamar Blue (12.5 mg resazurin dissolved in 100 mL phosphate buffered saline) were added to each well and the plates were incubated for another 2 h. Then the plates were read with a Spectramax Gemini XS microplate fluorometer as described for the leishmanicidal assay. Podophyllotoxin was used as standard drug.

## 4. Conclusions

Among the three most active antileishmanial compounds (**3**, **7** and **15**) only the cinnamic *N*-acylhydrazone derivative (**3**) present an excellent selectivity index (>42) (in only one case it was not cytotoxic against L-6 rat myoblast cells). *In vivo* leishmanicidal efficacy of this compound is being investigated and it will be described in due course. The results obtained for the trypanocidal activity against *T. brucei* rhodesiense were not encouraging (in one case the most active *N*-acylhydrazones were cytotoxic against L-6 rat myoblast cells).
